# Smoothed *l*_0_ Norm Regularization for Sparse-View X-Ray CT Reconstruction

**DOI:** 10.1155/2016/2180457

**Published:** 2016-09-20

**Authors:** Ming Li, Cheng Zhang, Chengtao Peng, Yihui Guan, Pin Xu, Mingshan Sun, Jian Zheng

**Affiliations:** ^1^Medical Imaging Department, Suzhou Institute of Biomedical Engineering and Technology, Chinese Academy of Sciences, Suzhou 215163, China; ^2^PET Center, Huashan Hospital, Fudan University, Shanghai 200235, China

## Abstract

Low-dose computed tomography (CT) reconstruction is a challenging problem in medical imaging. To complement the standard filtered back-projection (FBP) reconstruction, sparse regularization reconstruction gains more and more research attention, as it promises to reduce radiation dose, suppress artifacts, and improve noise properties. In this work, we present an iterative reconstruction approach using improved smoothed *l*_0_ (SL0) norm regularization which is used to approximate *l*_0_ norm by a family of continuous functions to fully exploit the sparseness of the image gradient. Due to the excellent sparse representation of the reconstruction signal, the desired tissue details are preserved in the resulting images. To evaluate the performance of the proposed SL0 regularization method, we reconstruct the simulated dataset acquired from the Shepp-Logan phantom and clinical head slice image. Additional experimental verification is also performed with two real datasets from scanned animal experiment. Compared to the referenced FBP reconstruction and the total variation (TV) regularization reconstruction, the results clearly reveal that the presented method has characteristic strengths. In particular, it improves reconstruction quality via reducing noise while preserving anatomical features.

## 1. Introduction

X-ray computed tomography has been widely used clinically for disease diagnosis, surgical guidance, perfusion imaging, and so forth. However, the massive X-ray radiations during CT exams are likely to induce cancer and other diseases in patients [1, 2]. Therefore, the issue of low-dose computerized tomography reconstruction has been raised and attracted more and more research attention. As far as we know, there are two low-dose strategies widely studied for dose reduction: (1) lowering X-ray tube current values, measured by milliampere (mA) or milliampere-seconds (mAs), or lowering X-ray tube voltage, measured by kilovolt (KV), and (2) lowering the number of sampling views during CT inspection. The strategy of regulation by mA or KV usually produces high noisy projection data. Thus, when the exposure dose is reduced, the images reconstructed using methods such as FBP suffer from increased artifacts and noise [3]. Diagnostic mistakes may appear in this case. The latter approach may also induce image artifacts due to limited sampling angles. As a result, the diagnostic value of the reconstructed images may be greatly degraded if inappropriate reconstruction approaches are applied.

To solve these problems, statistical reconstruction algorithms [4–9] attempt to produce high quality images by better modeling the projection data and the imaging geometry, which have shown superior performance compared to FBP-type reconstructions. Another path has been recently opened by compressed sensing (CS) with existing range of applications in medical imaging, for example, magnetic resonance imaging (MRI), bioluminescence tomography, optical coherence tomography, and low-dose CT reconstruction [10–24]. The CS theory reveals the potential capability of restoring sparse signals even if the Nyquist sampling theorem cannot be satisfied. Although the restricted isometry property (RIP) condition is not often satisfied in practice, CS-based reconstruction can yield more satisfying results than the traditional FBP algorithms in CT reconstruction [25]. Among several choices of sparse transforms, the gradient operator is motivated by the assumption that a preferable solution should be of bounded variation. It is known as total variation (TV) regularization, which favors solutions to be predominantly piecewise constant. TV has been widely used in the CT reconstruction community. However, TV-regularized images may suffer from loss of detail features and contrast, resulting in the staircasing artifacts. It is well known that *l*_0_ norm regularization can provide a sparser representation than the TV regularization (*l*_1_ norm) [26, 27]. However, the application of *l*_0_ norm in image reconstruction is often a nondeterministic polynomial-time (NP) hard problem. In addition, *l*_0_ norm is a nonconvex function in discontinuous form.

*l*_0_ norm is defined as the total number of its nonzeros elements and has stronger effects in promoting sparse solutions, but this minimization issue is NP hard to solve in general. Then, a spontaneous question can be whether preferable results will be achieved if we use regularization forms between *l*_1_ norm and *l*_0_ norm. In this work, we present a smoothed *l*_0_ (SL0) norm regularization model for sparse-view X-ray CT reconstruction. This SL0 regularization permits a dynamic regularization modulation and can achieve a good balance between the regularizations based on *l*_1_ norm and *l*_0_ norm. The paper is organized as follows. In Section 2, the SL0 norm model is firstly described and then the detailed optimization algorithm and the parameters setting are given.  Section 3 includes the experiments conducted on the projection data from the Shepp-Logan phantom, the head slice image, and the scanned mouse. The reconstructed results demonstrate that the proposed SL0 regularization produces better images with legible anatomical features and preferable noise characteristic compared to those using TV regularization. Finally, the discussions and conclusions are given at the end of this paper.

## 2. Methods

### 2.1. Problem Formulation

The idea of SL0 norm originates from the effort of minimizing a concave function that approximates *l*_0_ norm [26]. In order to address the discontinuity of *l*_0_ norm, we then try to approximate this discontinuous function via a feasible continuous one and minimize it by means of a minimization algorithm for continuous functions (e.g., steepest decent method). The continuous function which is used to approximate *l*_0_ norm should have a modulation parameter (say *σ*), which determines approximation degree. Then the family of the cost functions is defined as

(1) $$\begin{array}{c} f_{\sigma }\left(\;s\;\right)=1-e^{-\left|s\right|/2\sigma }, \end{array}$$

noting that

(2) $$\begin{array}{c} \underset{\sigma \to 0}{\lim}\;f_{\sigma }\left(\;s\;\right)=\left\{\begin{array}{cc} 0 & \text{if }s=0\\ 1 & \text{if }s\neq 0, \end{array}\right. \end{array}$$

or it can be approximately expressed as

(3) $$\begin{array}{c} f_{\sigma }\left(\;s\;\right)\approx \left\{\begin{array}{cc} 0 & \text{if }\left|s\right|\ll \sigma \\ 1 & \text{if }\left|s\right|\gg \sigma . \end{array}\right. \end{array}$$

Then SL0 norm is defined as

(4) $$\begin{array}{c} F_{\sigma }\left(\;s\;\right)=\overset{N}{\underset{i=1}{\sum }}f_{\sigma }\left(\;s_{i}\;\right). \end{array}$$

In (4), *N* is the length of reconstructed signals. From (2) and (3), we can obviously observe that when *σ* → 0, the SL0 norm tends to be equivalent to *l*_0_ norm. Therefore, we can find the minimal *l*_0_ norm solution via minimizing *F*_*σ*_(*s*) (subject to *As* = *p*) with a very small *σ* value. As can be seen, the value of *σ* determines the smoothness of the function *F*_*σ*_(*s*). The larger the value of *σ* is, the smoother *F*_*σ*_ is, resulting in worse approximation to *l*_0_ norm; and the smaller the value of *σ* is, the closer the performance between *F*_*σ*_ and *l*_0_ norm is.

Now, we recall the total variation (TV) norm of a 2-dimensional array (*x*_*i*,*j*_),  1 ≤ *i*, *j* ≤ *n*, which is defined as *l*_1_ norm of the magnitudes of the discrete gradient:

(5) $$\begin{array}{c} {\left\|\;x\;\right\|}_{TV}=\underset{i,j}{\sum }\left\|\;{\left(\;Dx\;\right)}_{i,j}\;\right\|, \end{array}$$

where (*Dx*)_*i*,*j*_ = (*x*_*i*+1,*j*_ − *x*_*i*,*j*_, *x*_*i*,*j*+1_ − *x*_*i*,*j*_); *x* is the attenuation coefficients to be reconstructed. If we use the proposed SL0 norm to enhance the sparsity of the image gradient, then the superior reconstruction behavior may be achieved. Therefore, to reconstruct the discrete X-ray linear attenuation coefficients, we consider the following constrained optimization problem:

(6) x∗=arg minx⁡ Fσx=arg minx⁡ ∑i,j1−e−Dxi,j/2σ,s.t. Ax−p≤ε, xi,j≥0,

where *A* is the system matrix, used to model the CT imaging system; *p* is the log-transformed projection measurements; *ε* is the tolerance used to enforce the data fidelity constraint, and it refers to X-ray scatter, electronic noise, scanned materials, and a simplified data model. Sidky and Pan [11] have indicated that the best image root-squared-error is achieved when chosen *ε* is around the actual error in the projection data. In practice, the real noise level of a system is usually unknown. Therefore, the optimal value of *ε* is selected when the reconstructed image with less artifacts and clearer anatomical structures is achieved.

### 2.2. Optimization Algorithm

In order to address the optimal solution of the proposed minimization problem, we try to assess the optimality of the solutions by analyzing the Karush-Kuhn-Tucker (KKT) conditions of (6) [28], which are the necessary conditions for optimality in nonlinear programming and can be derived through Lagrangian theory:

(7) x∗arg minx⁡ Lx,λ,η=arg minx⁡ Fσx+λAx−p−ε−∑i=1nηixi,

and the partial derivative of the above Lagrangian function can be expressed as

(8) ∂Lx,λ,η∂x=0⟺∇Fσx+λATAx−pAx−p−η⃑=0,

where the complimentary slackness is

(9) λAx−p−ε=0,ηixi=0

and the nonnegativity is

(10) λ≥0,ηi≥0.

In conclusion, the optimal solutions can be firstly satisfied with the projection data fidelity constraint, and then corresponding *λ* should satisfy *λ* > 0. Meanwhile, we intend to acquire the nonzero values of *x*_*i*_, and then corresponding *η*_*i*_ should satisfy *η*_*i*_ → 0. To obtain the solutions meeting the above conditions, we need to solve the following optimization problem:

(11) η∗=arg min⁡∇Fσx+λATAx−pAx−ps.t. λ>0.

Sidky and Pan [11] present an optimization approach composed by an iterative projection operator called projection-onto-convex-sets (POCS) and adaptive steepest descent procedure, which is suitable for dealing with large size constrained optimization problems. In this paper, a similar strategy is applied here. We choose POCS to be the iterative operator, which is an efficient iterative algorithm that can find images that satisfy the given convex constraints. POCS combines the ART technique and the image nonnegativity enforcement, and the proposed SL0 regularization is minimized via an iterative gradient descent of the cost function. The images are updated sequentially through the alternation of the POCS and gradient descent until the Karush-Kuhn-Tucker (KKT) conditions are satisfied. In practice, in order to reduce the computation time, we relax the KKT conditions or stop after a predefined iterative number. Under the current version of the proposed reconstruction algorithm, there is no rigidly theoretical proof on the convergence properties of the optimization procedure. However, the reconstructed results in the following experiments show that they are actually close to the optimal solution.

### 2.3. Parameters Selection

**Figure 1:**
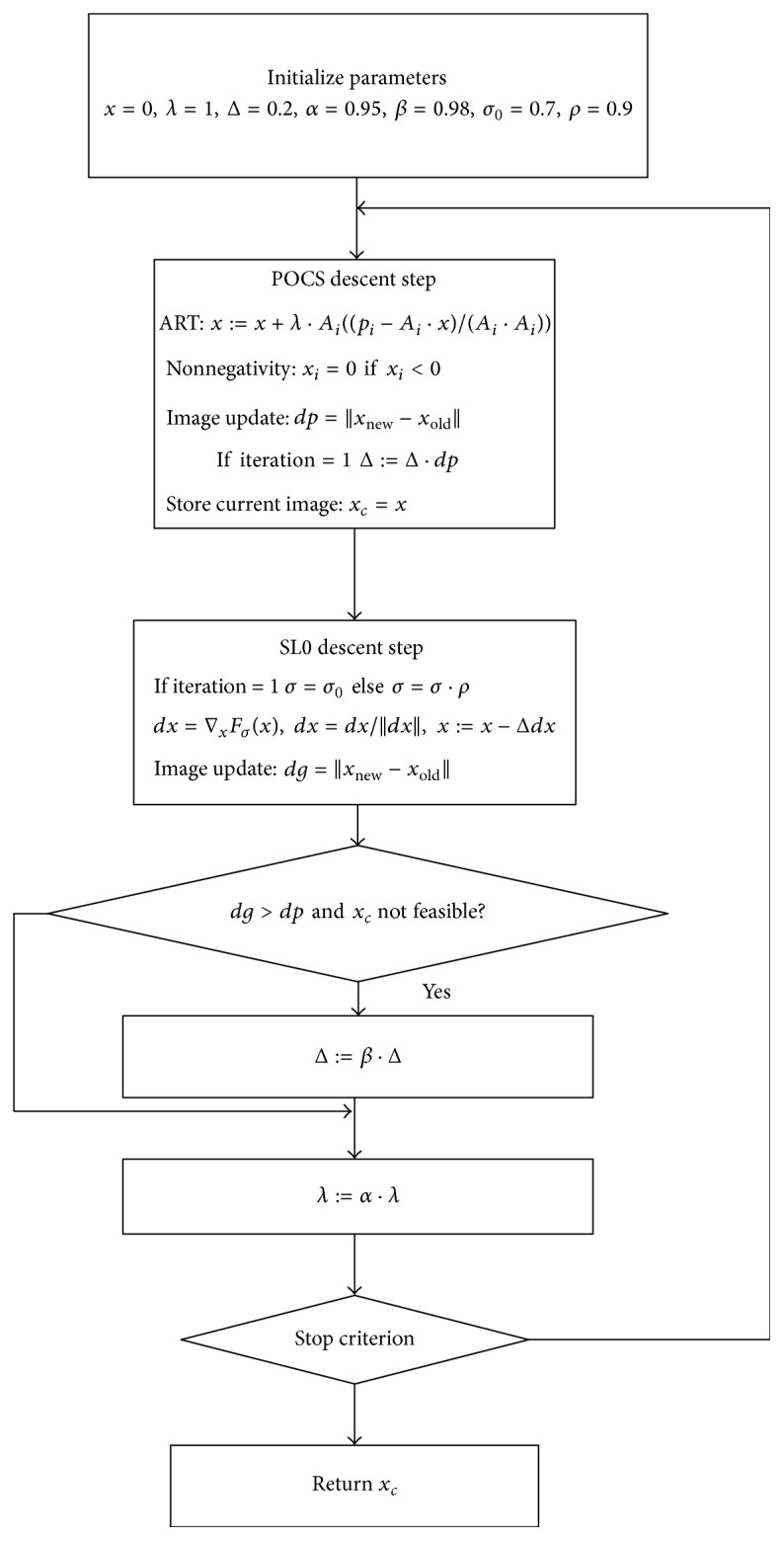
Flowchart of the proposed SL0 algorithm.

The implementation of the proposed SL0 regularization algorithm involves the choices of a series of parameters shown in Figure 1. The regularization parameter *σ* plays a crucial role in improving reconstruction quality. While we take a small value of *σ*, the function *F*_*σ*_ is highly unsmooth and includes many local minimums; hence finding its minimization is not easy. However, as *σ* increases, *F*_*σ*_ becomes smoother and includes less local minimums, and hence it is easier to minimize *F*_*σ*_. In general, if we use a larger value of *σ* during the whole iterative process, the smoother reconstruction results can be achieved but the tissue details are worse. On the other hand, if we use a smaller value of *σ* during the whole iterative process, the optimization process may get trapped into local minimum, which will lead to artifacts and noisy reconstructions. Hence, our idea is to solve a sequence of optimization problems. At the first step, we solve (6) using a larger value of *σ* (such as *σ*_0_). Subsequently, we reduce *σ*_0_ by multiplying a small factor *ρ* and then solve (6) again using *σ*_1_ = *ρσ*_0_. This time we initialize the reconstruction acquired in the last iteration. Due to the fact that *σ* decreases gradually, for each value of *σ*, the minimization algorithm starts with an initial solution close to the previous optimal value of *F*_*σ*_ (this is because both *σ* and *F*_*σ*_ have only slightly varied and consequently the minimization of new *F*_*σ*_ is potentially close to previous *F*_*σ*_). Hence, it is sufficient that the optimization algorithm is capable of escaping from getting trapped into local optimality and reaching the real minimum value for the small *σ* values, which offers the proximate *l*_0_ norm solution. In our tests, we select *σ*_0_ = 0.7 and *ρ* = 0.9 for all cases studied in this work. At the same time, the selection of *σ* should satisfy *σ*_min_ ≥ 0.01.

The parameters that control ART and the steepest gradient descent of objective function involve ART relaxation factor *λ*, which starts at 1.0 and slowly decreases to 0 as the iteration progresses; the steepest gradient descent relaxation factor Δ starts at 0.2 and slowly decreases to 0 as the iteration progresses. The decreasing factors *α* and *β* are the keys to control the respective step lengths for ART and SL0 steepest descent. In the following experiments, we select *α* = 0.95 and *β* = 0.98. The stopping criterion is reached if ‖*x* ^*k*+1^ − *x* ^*k*^‖_2_/‖*x* ^*k*^‖_2_ < 0.01 or the iterative process is stopped after a predefined maximum iteration number. In this paper, the maximum iterations of POCS are set to 30 and the maximum iterations of SL0 steepest descent are set to 20.

The above values are determined via experimental results, but we do not guarantee them to be optimal. However, the test results below demonstrate that the above parameters are satisfactory.

## 3. Experiments and Results

### 3.1. Data Acquisition

**Figure 2:**
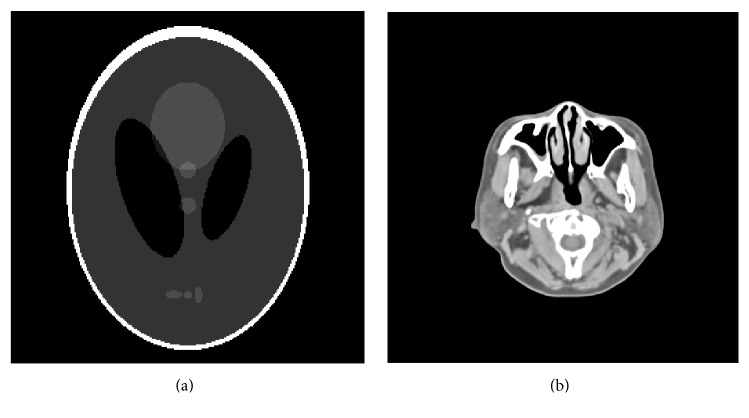
From left to right are Shepp-Logan phantom and human head slice image, respectively, which are the ground truth for reconstructions comparison. And the display windows are [1.0  1.1] and [−200  200] HU, respectively.

In order to characterize the superiority of the proposed SL0 regularization, we first study the performance of the proposed constrained optimization using the Shepp-Logan phantom and human head slice image. We used the Shepp-Logan phantom *I*: [0,256]×[0,256]→[0,2] with several ellipses standing for various anatomical tissues (see Figure 2(a)). The phantom was forward projected by MATLAB's radon routine with 720 projections over 2*π* rotation, yielding an angular spacing of 0.5°. The second sample dataset was a human head slice obtained from a clinical diagnostic CT device in our cooperative hospital (see Figure 2(b)). The projection data were generated according to the fan-beam CT geometry. The forward projection parameters were defined as follows: the source-to-axis distance was 42.5 cm and the distance of source-to-detector was 82.1 cm. The projection data of each view included 874 bins and the size of each element was 0.5 mm × 0.5 mm. And a total of 720 views were simulated during 2*π* rotation. The images to be reconstructed were composed by 512 × 512 pixels with 0.4 mm × 0.4 mm. Furthermore, in order to evaluate the performance under noisy projection data, we simulated the noisy measurements according to the following model [29, 30]:

(12) Ii=PoissonI0·exp⁡−∫Liμl,Ekdl+Normal0,σe2,

where *I*_*i*_ was the measured X-ray intensity in bin *i* and *I*_0_ was the incident intensity. *μ*(*l*, *E*_*k*_) was the energy-dependent attenuation map; *σ*_*e*_^2^ was the background electronic noise variance. In the simulation, we selected *I*_0_ = 5.0 × 10^5^ and *σ*_*e*_^2^ = 10. A monochromatic spectrum was assumed and the photon energy was set to 80 keV. Then the noisy projection data were obtained via logarithm transform.

In the second study, we evaluate the performance using two actual datasets from the scanned mouse experiments in our lab. The X-ray tube voltage and tube current were set to 50 kV and 1 mA, respectively. The projection data were acquired under fan-beam mode. The distance between the detector and the center of rotation was 436.6 mm, while the source-to-axis distance was set to 221.9 mm. A total of 360 projections were acquired over 2*π* rotation. The number of radial bins per view was 880, and the size of each bin was 0.15 × 0.15 mm^2^. The reconstructed image size was 512 × 512 with an isotropic pixel size of 95.7 *μ*m^2^.

### 3.2. Results

**Figure 3:**
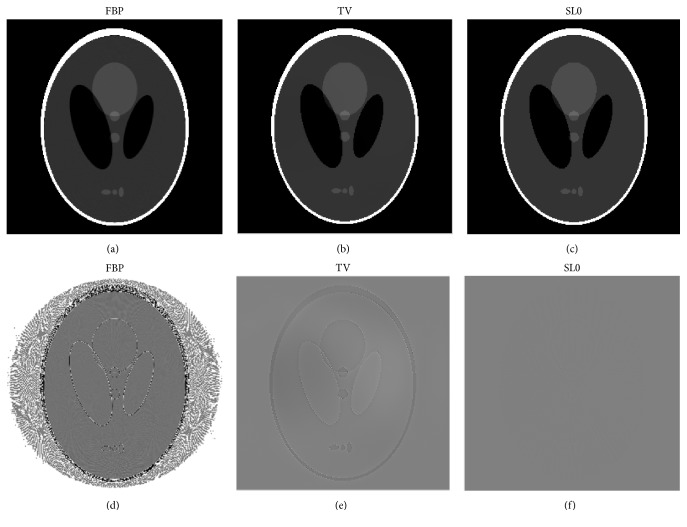
The results of Shepp-Logan phantom study. In (a), (b), and (c), from left to right, the reconstruction images are FBP, TV regularization, and SL0 regularization, respectively. And the display window is [1.0  1.1]. In (d), (e), and (f), the difference images between FBP, TV, and SL0 reconstructions and the ground truth are shown. And the display window is [−0.01  0.01].

We first start our evaluation with the Shepp-Logan phantom dataset, where the ground truth image is available. The images of the reconstruction are shown in Figure 3, where (a), (b), and (c) are for FBP, TV regularization, and SL0 regularization, respectively. Among them, FBP is applied to the entire projection data. However, we only select 120 views (equally spaced over 2*π* rotation) for TV regularization and SL0 regularization. As can be seen in (a), (b), and (c) in Figure 3, we cannot observe significant difference between the reconstructions. In order to make the otherness of reconstructed results highlighted, the differences between the reconstructed images and the original image (OI) of the Shepp-Logan phantom are calculated. We can see in Figure 3 ((d), (e), and (f)) that the proposed SL0 regularization algorithm leads to the best image quality with effectively preserved margin details.

**Figure 4:**
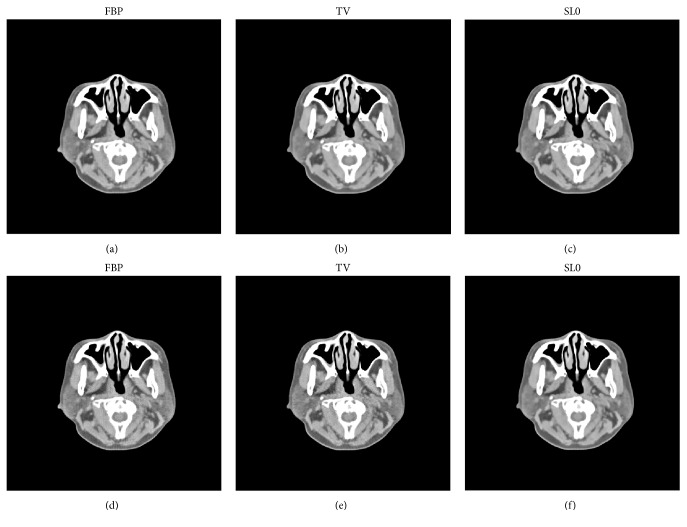
The results of human head slice simulation study. In (a), (b), and (c), from left to right, the reconstruction images are FBP, TV regularization, and SL0 regularization using noiseless projections. And the display window is [−200  200] HU. In (d), (e), and (f), the corresponding reconstructed images with simulated noisy projection data are shown. And the display window is [−200  200] HU.

**Figure 5:**
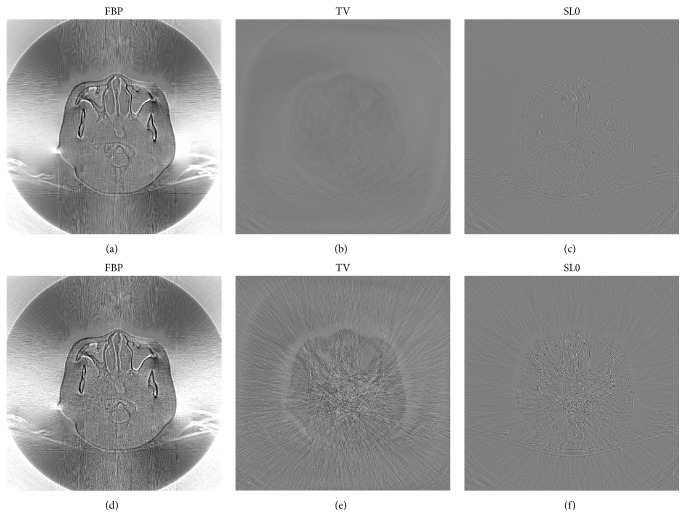
The difference between the reconstructed image and the original image (OI) of the human head slice. From top to bottom, there are noiseless and simulated noisy scenarios in turn. From left to right, the reconstruction algorithms are FBP, TV, and SL0, respectively. The display window is [−70  70] HU.

For the head slice dataset, the reconstructed images are shown in Figure 4 for all three reconstruction methods. The total of 720 views is completely selected for FBP reconstruction, and only 180 views of them are used for TV and SL0 regularization reconstruction. (a), (b), and (c) in Figure 4 illustrate the reconstructed results through FBP, TV, and SL0 using noiseless projections. Compared to the head slice sample, FBP reconstruction produces obvious image artifacts, but TV and SL0 reconstructions well reflect the sample image even with apparently undersampled measurements. (d), (e), and (f) in Figure 4 show the reconstructed results through FBP, TV, and SL0 using simulated noisy projections. When compared to the head slice sample, FBP and TV reconstructions introduce significant artifacts and the images appear to be very noisy. In this case, SL0 is superior to FBP and TV with vastly suppressed artifacts and better preserved image structures. Furthermore, we also compute the difference between the reconstructed image and the original image (OI) of the human head slice and the results are illustrated in Figure 5. It can be observed from Figure 5 that the SL0 produces minor differences between the reconstructed images and the reference image when compared to those of FBP and TV, which agrees with the observations from Figure 4.

To further quantify the performance of the proposed SL0 method with FBP and TV methods, there are two criterions to evaluate the reconstructed image. One is the normalized mean absolute deviation (NMAD), defined as

(13) $$\begin{array}{c} NMAD\left(\;\%\;\right)=\frac{\sum_{i,j}\left|x_{i,j}-x_{i,j}^{truth}\right|}{\sum_{i,j}\left|x_{i,j}^{truth}\right|}\times 100. \end{array}$$

And the other one is the signal-to-noise ratio (SNR), defined as

(14) $$\begin{array}{c} SNR=10\times \mathrm{lg}\left(\;\frac{\sum_{i,j}{\left(x_{i,j}^{truth}\right)}^{2}}{\sum_{i,j}{\left(x_{i,j}-x_{i,j}^{truth}\right)}^{2}}\;\right). \end{array}$$

The values of the two criterions are presented in Table 1. Among these three algorithms, FBP produces the worst results with highest NMADs and lowest SNRs. In Shepp-Logan phantom experiments, both TV and SL0 generate the superior performances with teeny NMADs, which indicate that the reconstructions are comparatively close to the ground truth. In head slice image experiments, the quality of all the reconstructions is decreased with the simulated Poisson noise. However, in comparison to FBP and TV, SL0 generates the optimal results under all the situations, which are consistent with the observations in Figures 3, 4, and 5.

**Figure 6:**
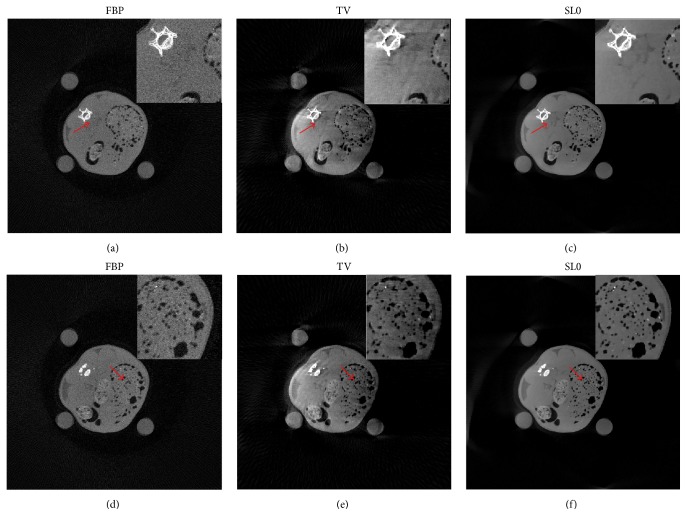
Results of scanned mouse datasets. From top to bottom, there are two different slices reconstructions. From left to right, the reconstruction images are FBP, TV regularization, and SL0 regularization, respectively. The red arrows denote a small area of interest and corresponding zoomed images of ROI are placed at the top right. And the display window is [0  1.5].

Finally, in Figure 6, we present the reconstructed results for scanned mouse data. The whole projection data are chosen for FBP reconstruction and only half of them are used for TV and SL0 regularization reconstruction. The reconstruction images are shown in Figure 6 for all the three reconstruction algorithms. A small area of interest is highlighted with a magnification factor of 2, and the zoomed images of this region are shown in the corresponding upper right corner. As can be seen, severe noise can be observed in the FBP results and the images appear to be blurry near to margin details. Compared to FBP, better preserved soft tissue edges and obviously reduced noise level can be observed in TV results. We can see in Figure 6 that the proposed SL0 method leads to the significantly improved image quality with effective noise suppression and tissue structure preservation in comparison to FBP and TV.

## 4. Discussion

In this paper, we propose smoothed *l*_0_ norm optimization algorithm that exploits the gradient sparseness for low-dose CT imaging. The results demonstrate that the proposed method can effectively reduce noise and produce significantly improved images. Compared to TV regularization method, it is advantageous in terms of improved tissue edge properties, as well as lower level artifacts and image noise. The approximation of *l*_0_ norm scheme via a family of continuous functions allows us to fully exploit the sparse assumption imposed on image gradient (IG) and generate a feasible method for sparse-view CT reconstruction.

**Figure 7:**
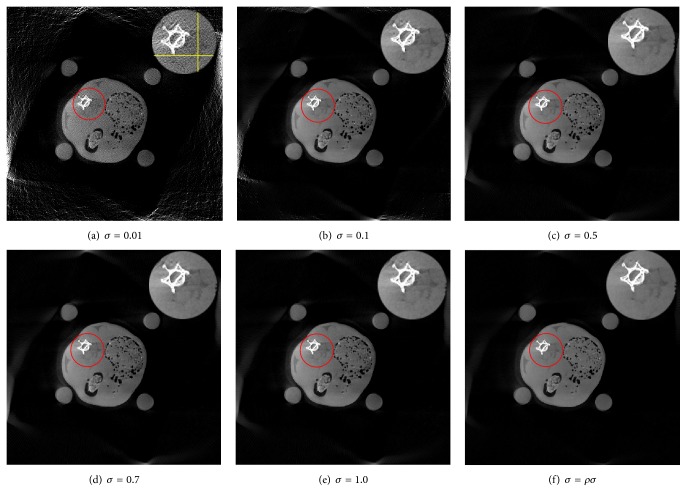
Results of reconstruction using different *σ* parameters. (a)–(e) are the reconstructions with singular *σ* value during the whole iterative process. (f) is the reconstructed image with the decreased *σ* value. The red circles denote a small area of interest and corresponding zoomed images of ROI are placed at the top right. The initial *σ* equals 0.7 and *ρ* equals 0.9. And the display window is [0  1.5].

**Figure 8:**
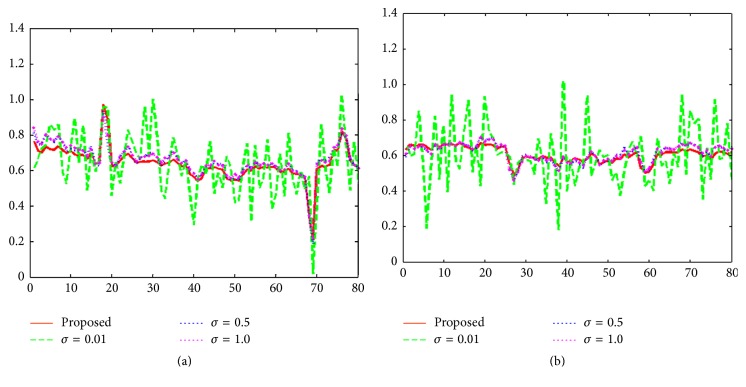
Line profiles of ROI in Figure 7. (a) Transversal profiles of ROI. (b) Vertical profiles of ROI.

The sequentially updated *σ* values originate from the effort to find a measure that better approximates *l*_0_ norm than the traditional TV regularization method (*l*_1_ norm). By altering parameter *σ*, we can obtain better control of the IG sparsity, which produces the superior anatomical features over the TV minimization. The regularization parameter *σ* plays a vital role in improving reconstruction quality. In order to acquire the better *σ* selection, we perform a series of reconstruction experiments with different *σ* values. As can be seen through Figures 7(a)–7(e), when we take *σ* = 0.01, the cost function *F*_*σ*_ tends to give the closer behavior to *l*_0_ norm, but the reconstructed image is the worst with severe artifacts and noise. However, as *σ* increases, the reconstruction images appear to improve gradually with obviously reduced noise level. In Figures 7(a)–7(e), we can also observe that the reconstructions with singular *σ* value during the whole iterative process cannot adequately suppress artifacts and preserve tissue structures (see the regions indicated by the red circles). In order to obtain the preferable reconstruction, the motivation of solving a sequence minimization strategy through orderly decreased *σ* value seems to be a suitable choice if both artifacts and noise suppression and margin details preservation are pursued. In the test, we select the initial value of *σ* as 0.7 and the decreased factor *ρ* as 0.9. In Figures 7(a)–7(f), we can clearly observe that the sequential optimization via *σ* = *ρσ* can lead to the optimal image quality with effectively suppressed artifacts and significant improved edge properties. Additionally, we also show line profiles along the marked yellow lines for ROIs of *σ* = 0.01, 0.5, and 1.0 and proposed scenarios in Figures 8(a) and 8(b). It can be observed from Figure 8 that the proposed *σ* selection can produce image with less artifact and noise, which agrees with the observation in Figure 7.

A limitation of the proposed SL0 approach lies in the sparsity assumption on the IG, which is an ordinary problem for all the sparsity-driven iterative methods in CT reconstruction. For most numerical or physical phantoms, the reconstructed images are piecewise smooth and the sparsity assumption on the IG is valid. However, this will affect SL0 for human or animal slice reconstruction when images only have a merely low level of sparseness on the IG. Fortunately, the parameter *σ* allows us to expediently control the aggressiveness in encouraging sparsity with TV as *σ* regulates. Another potential problem is that when a 512 × 512 image is to be reconstructed, the SL0 algorithm takes around 65 s to finish one loop on a 2.67 GHz PC with 4 GB RAM under MATLAB R2011a. There are several ways to improve computational efficiency. One way is to select the conjugate gradient (CG) method to solve the reconstruction problems [28]. The CG algorithm is an improved steepest descent algorithm, with the descent direction determined by the current descent direction as well as the previous searching direction. In addition, the proposed algorithm can be accelerated via GPU-based technique to fulfill the clinical requirements [31].

## 5. Conclusion

In this work, we studied sparse regularization for X-ray low-dose CT imaging using a smoothed *l*_0_ norm (SL0) model. We investigated SL0 and compared its results with TV regularization and FBP on a numerical phantom and a clinical head slice as well as on two real datasets from scanned animal experiments. From the results, we have seen that the proposed SL0 regularization has yielded improved reconstructions with better performance in edge preservation and noise suppression compared to the other two methods. Nevertheless, practical application of the proposed approach still needs further validation using more actual clinical data. In the future, we will focus on addressing the limitations of our research described above. Furthermore, we will try to extend the SL0 regularization to handle other incomplete data reconstruction problems [32].

## Figures and Tables

**Table 1:** Comparing criterions of the results reconstructed by different algorithms (Shepp-Logan and head slice).

	FBP		TV		SL0	
	NMAD (%)	SNR (dB)	NMAD (%)	SNR (dB)	NMA (%)	SNR (dB)
Shepp-Logan phantom	1.23	36.49	3.6*e* − 02	67.70	6.1*e* − 04	93.87
Head slice image (noiseless)	3.13	28.09	0.27	47.68	0.25	49.98
Head slice image (Poisson)	3.36	27.75	0.95	38.47	0.47	42.20
